# Emergency Management of Obstructing Ureteric Stones: A Closed-Loop Audit of Hot Ureteroscopy Uptake Against Getting It Right First Time (GIRFT) Standards

**DOI:** 10.7759/cureus.116674

**Published:** 2026-09-21

**Authors:** Ayman E Girgis

**Affiliations:** 1 Urology, Kettering General Hospital, Kettering, GBR; 2 Urology, George Eliot Hospital, Nuneaton, GBR

**Keywords:** emergency, girft, hot ureteroscopy, obstructing, ureteric stones

## Abstract

Introduction and aim: National guidance recommends hot (same-admission) ureteroscopy and laser lithotripsy as the default definitive treatment for suitable obstructing ureteric stones, rather than defaulting to temporizing measures such as ureteric stent insertion. The primary aim was (1) to determine the proportion of emergency ureteric stone cases managed by hot ureteroscopy against the GIRFT standard. Secondary aims were (2) to describe the pattern of emergency management and identify deviations from evidence-based practice, and (3) to assess the impact of a targeted action plan on the hot ureteroscopy rate. In addition, this article reports emergent findings identified during data review, including inter-surgeon variation in hot ureteroscopy uptake and individual case-level governance considerations, which were not prespecified analyses but are presented descriptively for their quality-improvement value.

Methods: Cycle 1 (baseline) captured all emergency ureteric stone admissions over a three-month period (March to May 2024). Following presentation of results and an agreed action plan, cycle 2 (re-audit) captured all surgically-managed cases over the subsequent three-month period (August to October 2024). The primary outcome was the proportion of surgically-managed cases treated by hot ureteroscopy.

Results: Cycle 1 identified 48 admissions, of which 22 were managed surgically; hot ureteroscopy was performed in only five (22.7%), with 17 (77%) judged retrospectively suitable. Tamsulosin was prescribed for all conservatively-managed stones regardless of size or location, contrary to trial and guideline evidence discussed below. Following the action plan, cycle 2 identified 20 surgically-managed cases; hot ureteroscopy rose to 10 (50%), more than doubling cycle 1's rate. Seven of the 10 stented-only cases were retrospectively judged suitable for hot ureteroscopy; individual surgeon rates ranged up to 100%, against a department average of 50%.

Conclusions: A structured closed-loop audit with an explicit action plan more than doubled the hot ureteroscopy rate for obstructing ureteric stones, moving practice closer to national standards. Residual missed opportunities and surgeon-level variation support a dedicated hot ureteroscopy list and further audit cycles.

## Introduction

Ureteric colic secondary to obstructing ureteric stones is one of the most common urological emergencies encountered in UK hospitals, generating a substantial burden of unscheduled admissions, imaging, and theater workload. Historically, management of the acutely obstructed, infected, or symptomatic ureteric stone in many units has defaulted to temporizing ureteric stent insertion, followed by a separate elective admission for definitive stone treatment by ureteroscopy or extracorporeal shock wave lithotripsy (ESWL). This two-stage pathway exposes patients to two general anesthetics, a period with an indwelling stent, associated with substantial patient-reported morbidity including urinary frequency, dysuria, and flank pain, and a wait for definitive treatment [1].

The Getting It Right First Time (GIRFT) national specialty report, urology: towards better care for patients with acute urinary tract stones (January 2022), set out an explicit standard for acute stone services as follows: definitive treatment by lithotripsy or ureteroscopic surgery should be available to patients within an appropriate timescale, supported by smooth pathways to a fixed-site lithotripter and access to a laser-enabled emergency operating theater with appropriately trained staff and surgeons [2]. This is complemented by National Institute for Health and Care Excellence (NICE) guidance on the assessment and management of renal and ureteric stones, which similarly frames the timeliness of definitive intervention as central to good care [3]. In practice, this means that hot (same-admission) ureteroscopy with laser lithotripsy should be considered the default surgical option for a suitable obstructing ureteric stone, rather than an exception reserved for cases with proven infection alone.

Alongside surgical decision-making, there is a well-established evidence base that questions the routine use of medical expulsive therapy (alpha-blockers such as tamsulosin) to facilitate spontaneous stone passage. While an earlier meta-analysis of smaller trials by Hollingsworth et al. suggested a benefit [4], the subsequent large, multicenter, placebo-controlled Spontaneous Urinary Stone Passage Enabled by Drugs (SUSPEND) trial found no significant difference in stone clearance between tamsulosin, nifedipine, and placebo [5], a finding now reflected in current European Association of Urology (EAU) guidance on urolithiasis [6]. Continued blanket prescribing of tamsulosin regardless of stone size or location therefore represents a further area where local practice may drift from the evidence base.

Prompted by informal impressions that hot ureteroscopy was being underused in our department relative to the GIRFT standard, we undertook a first audit cycle in July 2024 examining all emergency ureteric stone admissions over a three-month period. This identified a low hot ureteroscopy rate and a number of missed opportunities, and generated a specific, actionable improvement plan. A second cycle (re-audit) was carried out in October 2024 to evaluate the impact of that plan, consistent with the established principle that clinical audit delivers its greatest value when the cycle is closed by re-measurement against an agreed action plan, rather than concluding at the point of initial data collection [7]. This article was previously presented, in part, as a departmental audit report at the local urology audit meeting on July 31, 2024, and subsequently at a trust-wide teaching day on August 31, 2024. This article reports both cycles as a single closed-loop audit.

## Materials and methods

This was a retrospective, closed-loop, two-cycle audit conducted in a single urology department. Cycle 1 (baseline) and cycle 2 (re-audit) followed the same broad methodology of case identification and data extraction, with a change in scope for cycle 2 as described below.

Cycle 1: baseline audit (March to May 2024)

All cases of ureteric stones admitted throughout March, April, and May 2024 were identified using ward-level data (the departmental W-Drive) and the trust clinical portal. Re-admissions for the same stone episode were excluded to avoid double-counting. For each case, data were extracted on patient demographics (age, sex, American Society of Anesthesiologists {ASA} grade), stone characteristics (size and ureteric location {proximal, middle, or distal third}), and the emergency management pathway received (conservative management with or without medical expulsive therapy, nephrostomy insertion, ureteric stent insertion, hot ureteroscopy, delayed primary ureteroscopy, or extracorporeal shock wave lithotripsy {ESWL}). Where conservative management was pursued, radiologically-confirmed stone passage was recorded.

Cycle 2: re-audit (August to October 2024)

Following an agreed action plan, cycle 2 examined all emergency ureteric stone cases that underwent surgical treatment between August and October 2024. Unlike cycle 1, which captured the full admission cohort including conservatively-managed cases, cycle 2 was scoped specifically to the surgically treated subgroup, since the primary purpose of the re-audit was to evaluate the impact of the action plan on surgical decision-making; this difference in scope is addressed in further text.

Standard and outcome measure

The principal standard against which practice was benchmarked was the Getting It Right First Time (GIRFT) (January 2022) recommendation that definitive stone treatment be available within an appropriate timescale via a laser-enabled emergency theater [2]. The primary outcome measure in both cycles was the proportion of surgically-managed cases treated by hot ureteroscopy. To allow direct comparison between the two cycles despite their differing scope, this proportion was calculated as a percentage of the surgically-managed subgroup in each cycle (22 cases in cycle 1; 20 cases in cycle 2), rather than as a percentage of all admissions. Retrospective suitability for hot ureteroscopy, reported for context alongside the primary outcome, was assessed by the author as a single, unblinded reviewer, applying GIRFT-aligned criteria: a non-infected obstructing stone in a patient with renal impairment, uncontrolled pain, and/or an expressed preference for definitive same-admission treatment. No prospectively validated selection instrument was used, and no second reviewer or inter-rater reliability check was performed. Data extraction for both cycles was similarly performed by a single reviewer, without second-reviewer cross-validation. Given the descriptive, quality-improvement design of this audit and the modest sample sizes involved, results are reported as proportions and percentages; no formal inferential statistical testing was planned or performed. As a retrospective service evaluation and clinical audit of existing standard care using anonymized departmental data, this project did not constitute research requiring formal ethics committee review, consistent with UK Health Research Authority guidance on audit.

## Results

Cycle 1: case volume, demographics, and stone characteristics

A total of 48 cases of ureteric stones were identified over the following three-month period: 14 in March, 25 in April, and nine in May. Patient age ranged from 22 to 79 years. The cohort included 27 female and 21 male patients. The majority of patients were American Society of Anesthesiologists (ASA) grade 1. Stone size ranged from 2 mm to 1.3 cm (2 mm, n=2; 3 mm, n=5; 4 mm, n=9; 5 mm, n=8; 6 mm, n=10; 7 mm, n=4; 8 mm, n=4; 9 mm, n=3; 11 mm, n=1; 13 mm, n=2). Stone location was proximal ureter in 21 cases, mid-ureter in one case, and distal ureter in 26 cases.

Cycle 1: emergency management pathway

Of the 48 cases, 26 (54%) were managed conservatively with analgesia and observation, with radiologically-confirmed stone passage in 80% of this group. Tamsulosin was prescribed in every one of these 26 cases irrespective of stone size or location, a practice not supported by the evidence discussed above [4-6]. The remaining 22 cases were managed surgically or via a drainage procedure as follows: two nephrostomy insertions (both for associated sepsis), 12 ureteric stent insertions (two for proven infection, eight with no trial of hot ureteroscopy), five hot ureteroscopies (including one septic 7 mm proximal stone), two delayed primary ureteroscopies (one managed conservatively initially despite a C-reactive protein of 121 mg/L and white cell count of 16×10^9^/L), and one ESWL (6.7 mm left ureteric stone, still awaited at the time of reporting) (Figure 1, Table 1).

**Figure 1:**
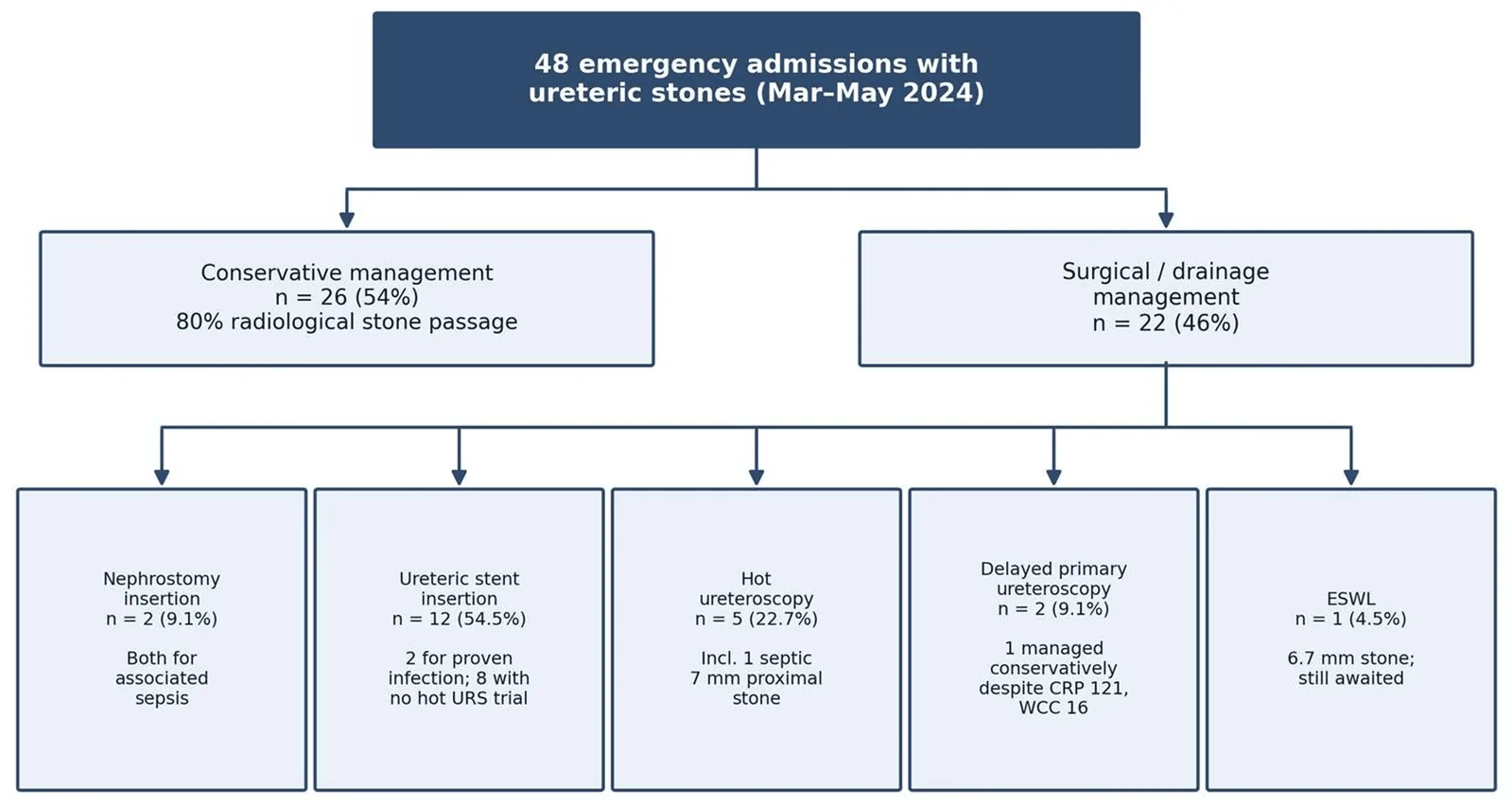
Emergency management pathway, cycle 1 (March to May 2024). Of 48 admissions, 22 were managed surgically or by drainage procedures; hot ureteroscopy accounted for only five cases (22.7% of the surgical subgroup), compared with a retrospectively estimated 17 (77%) suitable candidates. WCC: white cell count; ESWL: extracorporeal shock wave lithotripsy; URS: ureteroscopy

**Table 1:** Surgical/drainage management of the 22 non-conservatively-managed cases, cycle 1 (March to May 2024). WCC: white cell count; ESWL: extracorporeal shock wave lithotripsy

Management	Cases, n	Percentage of surgical subgroup (n=22)		Notes		
Nephrostomy insertion	2	9.1%		Both for associated sepsis		
Ureteric stent insertion	12	54.5%		2 for proven infection; 8 with no trial of hot ureteroscopy		
Hot ureteroscopy	5	22.7%		Including 1 septic 7 mm proximal stone		
Delayed primary ureteroscopy	2	9.1%		1 managed conservatively initially despite CRP 121 mg/L, WCC 16×10^9^/L		
ESWL	1	4.5%		6.7 mm left ureteric stone (March); still awaited at the time of reporting		

Eight of the 12 patients who received a ureteric stent had no documented trial of hot ureteroscopy, despite the absence of proven infection in most of these cases. Applying GIRFT-aligned criteria of a non-infected obstructing stone with renal impairment, uncontrolled pain, and/or patient preference, retrospective review suggested that 17 of the 22 surgically-managed cases (77%) would have been suitable candidates for hot ureteroscopy, against the five cases (22.7%) in which it was actually performed.

Cycle 1: action plan

Following presentation of these findings, the following actions were agreed: (1) adopt hot ureteroscopy and laser lithotripsy as the default surgical modality for non-infected obstructing ureteric stones in cases with renal impairment, uncontrolled pain, and/or patient preference; (2) reserve acute ESWL for cases with genuine access to fixed-site lithotripsy; (3) list stented/nephrostomized patients for elective ureteroscopy within four weeks, per GIRFT guidance; (4) consider a dedicated hot ureteroscopy theater list; and (5) name a clinician responsible for timely listing of cases for elective ureteroscopy.

Cycle 2: case volume and stone characteristics

A total of 20 cases were managed surgically for obstructing ureteric stones between August and October 2024: six in August, six in September, and eight in October. Stone size ranged from 4 mm to 1.8 cm; one case represented steinstrasse (a column of retained fragments obstructing the ureter) following prior ESWL. Stone location was distal ureter in five cases, mid-ureter in one case, and proximal ureter in 14 cases, representing a markedly higher proportion of proximal stones than in the cycle 1 surgical subgroup.

Cycle 2: hot ureteroscopy outcomes and practice variation

Hot ureteroscopy was performed in 10 of the 20 cases (50%) (Figure 2). Two cases were judged unsuitable for ureteroscopy because of associated infection. Five hot ureteroscopy cases involved proximal ureteric stones, the largest measuring 18 mm. Flexible ureteroscopy was required in two of the 10 hot ureteroscopy cases as follows: one because of stone retropulsion during the procedure, and the second for the 18 mm proximal stone, in which a stone-free outcome was subsequently confirmed radiologically on computed tomography. One hot ureteroscopy was performed without clear radiological evidence of a ureteric stone at the time of surgery, based on persistent pain and a remote history of renal stones; no stone was identified on either rigid or flexible ureteroscopy in this case.

**Figure 2:**
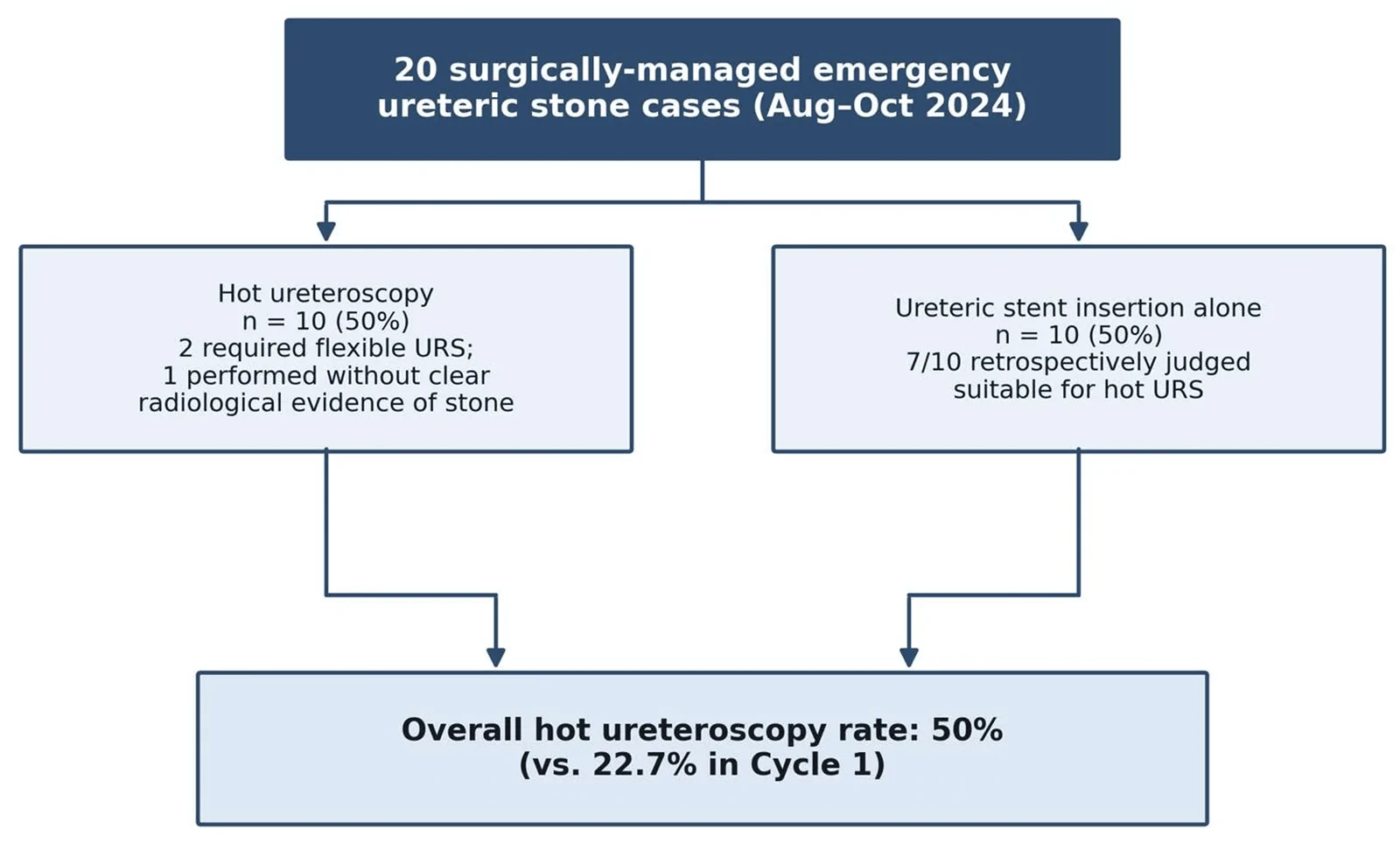
Emergency management pathway, cycle 2 re-audit (August to October 2024). Following the cycle 1 action plan, hot ureteroscopy rose to 10/20 (50%) of surgically-managed cases - seven of the 10 cases managed by stent alone were judged suitable for hot ureteroscopy. URS: ureteroscopy

A total of 10 cases were managed by ureteric stent insertion alone, with ureteroscopy attempted in one of these; retrospective review judged seven of these 10 stented cases to have been suitable for hot ureteroscopy. Marked variation in practice was identified between individual surgeons as follows: one surgeon achieved a 100% hot ureteroscopy rate across six cases, against a department-wide average of 50%. Overall, the hot ureteroscopy rate more than doubled between the two audit cycles, from 22.7% to 50% of surgically-managed cases (Figure 3, Table 2).

**Figure 3:**
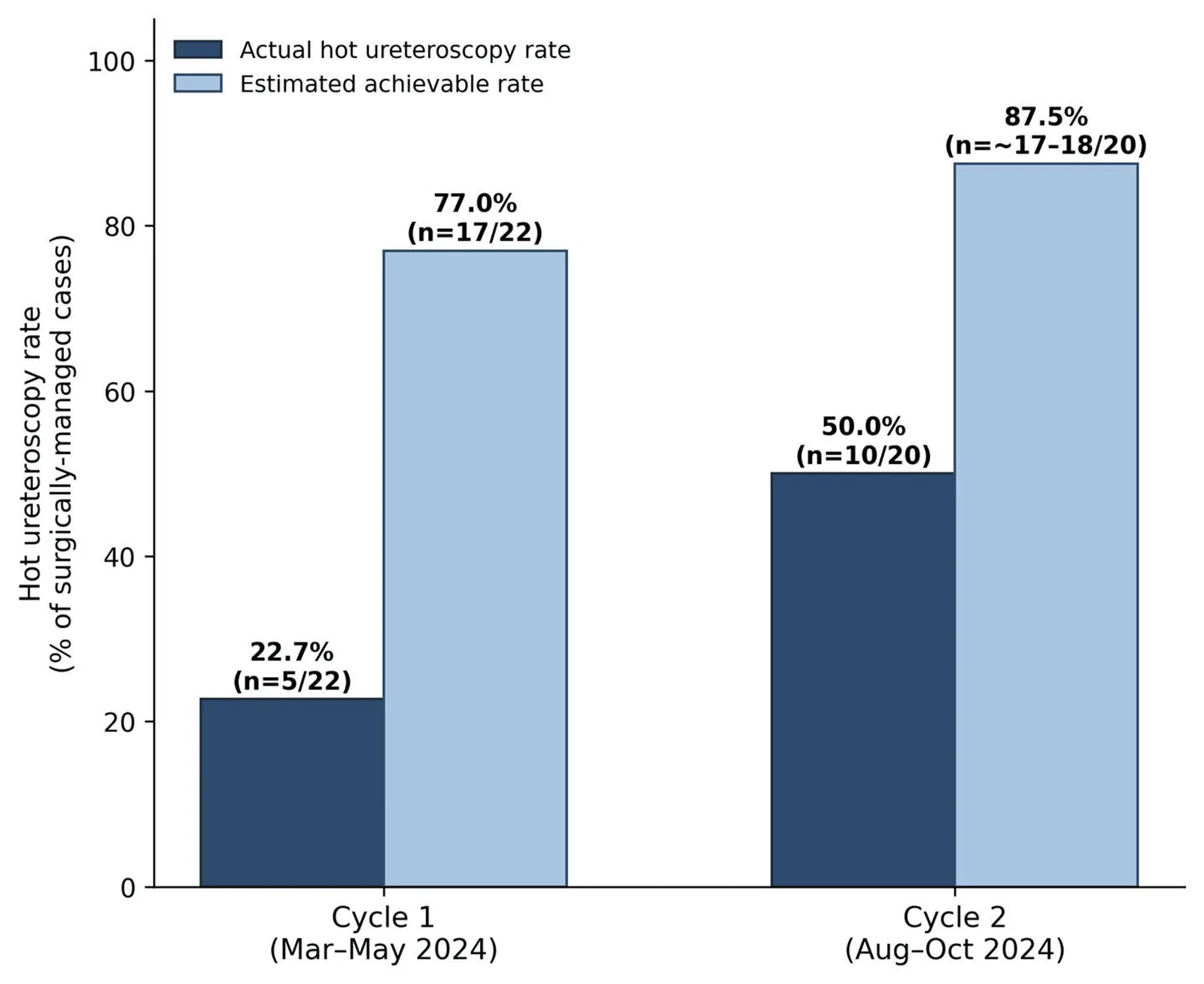
Hot ureteroscopy rate by audit cycle. Actual vs. estimated achievable hot ureteroscopy rate, as a percentage of surgically-managed cases in each cycle, with the underlying case count (n) shown alongside each percentage.

**Table 2:** Comparison of hot URS uptake between cycle 1 and cycle 2. URS: ureteroscopy

Metric			Cycle 1 (March-May 2024)		Cycle 2 (August-October 2024)
Surgically-managed cases (denominator), n			22		20
Hot ureteroscopy performed, n			5		10
Hot ureteroscopy rate, %			22.7		50
Cases managed by stent alone (no attempted hot URS), n			8		10
Retrospectively suitable for hot URS but not offered it, n			12 of 17 identified candidates		7 of 10 stented cases
Estimated achievable rate, %			~77 (17/22)		~85-90 (as estimated)

## Discussion

This closed-loop audit demonstrates a substantial, measurable improvement in adherence to the GIRFT (January 2022) standard for acute stone management following a single, clearly defined action plan [2]. The hot ureteroscopy rate more than doubled between cycles, from 22.7% to 50% of surgically-managed cases, without any change in the underlying patient population that would obviously explain the shift; indeed, cycle 2 contained a higher proportion of proximal stones, which are generally more technically demanding to treat via ureteroscopy than distal stones, making the improvement more notable rather than less. This comparison is of the proportion of surgically managed subgroups in each cycle (22 cases in cycle 1 and 20 in cycle 2), not of total admission volumes, which differed in scope between cycles as described in the earlier text. The increase was not tested for statistical significance given these small subgroup sizes and should be interpreted as a descriptive, service-level finding.

Despite this improvement, the audit identified continued missed opportunities. In cycle 2, seven of the 10 patients managed by stent insertion alone were judged retrospectively suitable for hot ureteroscopy, indicating that roughly a third of the total surgical cohort (7/20) could plausibly have received definitive same-admission treatment but did not. This finding is consistent with the marked variation in practice observed between individual surgeons, ranging from a 100% hot ureteroscopy rate for one surgeon to a department average of 50%. Such variation suggests that the limiting factor in many cases may relate less to patient suitability and more to theater access, out-of-hours staffing, individual surgeon confidence with flexible ureteroscopy and laser lithotripsy, or local custom and practice, consistent with the cycle 1 action plan's proposal for a dedicated hot ureteroscopy theater list and a named clinician responsible for timely case listing. This pattern is well recognized in the wider literature on barriers to same-admission stone intervention: McKay et al., in the same comparative study discussed above, explicitly identify access to emergency theaters, theater staff skills, theater facilities, and surgical endourological experience as the principal obstacles to expanding primary ureteroscopy provision [8]. Chislett and Qu, in a structured review of barriers across the acute ureteric colic pathway, similarly catalog theater and staffing constraints, out-of-hours surgical experience, and local service configuration as recurring, modifiable obstacles distinct from patient-level factors [9]. Blackwell et al., analyzing a large US administrative dataset, further demonstrated a measurable "weekend effect" in acute stone care, with patients admitted on weekends experiencing significant delays to intervention compared with weekday admissions, attributable to reduced out-of-hours theater and staffing availability rather than to differences in case complexity [10]. These findings are consistent with, though do not conclusively establish, an interpretation in which the inter-surgeon variation observed in cycle 2 reflects structural and organizational barriers at least as much as differences in individual clinical judgment. Case-level analysis of individual surgical decision-making was outside the scope of this audit, reinforcing the case for the service-level solutions, a dedicated list and named responsible clinician, proposed in our action plan, rather than relying on individual surgeon initiative to overcome what is, in essence, a systems problem.

Our findings sit within the growing body of comparative evidence favoring earlier, same-admission intervention. Khopekar et al., in a UK quality-improvement study, found that reducing the interval between CT detection and ureteroscopic laser fragmentation achieved clinical outcomes comparable to delayed, stent-first management while being substantially more cost-effective, averting an estimated £2,868 per patient for the health system by avoiding the costs of a second admission and procedure [11]. Similarly, Al-Terki et al., comparing emergency with elective ureteroscopy for a single ureteric stone, reported comparable stone-free rates between the two approaches, supporting the safety of an emergency-first strategy in appropriately selected patients [12]. McKay et al. reached a comparable conclusion in a prospective non-randomized comparison of primary vs. delayed ureteroscopy, finding similar safety and efficacy outcomes between the two timing strategies [8]. This is further reinforced by a more recent systematic review and meta-analysis by Alsawi et al., pooling 12 studies and almost 2,700 patients, which found primary ureteroscopy to be associated with significantly lower subsequent stent usage, equivalent stone clearance, and no increase in overall or major complications, including sepsis, compared with delayed ureteroscopy, with only a modest reduction in minor complications favoring the delayed approach [13]. Wani et al. similarly found primary ureteroscopy to be both clinically and economically favorable compared with a default emergency-stenting strategy, reinforcing the case for hot ureteroscopy as a default rather than a selectively applied option [14]. Taken together, this literature supports our department's action-plan position that hot ureteroscopy should be the default rather than the exception for suitable stones, and suggests that the missed opportunities identified in cycle 2 carry a measurable cost, both to patients, in the form of avoidable second admissions and prolonged stenting, and to the health system, in the form of the additional procedure and bed-day costs quantified by Khopekar et al. [11].

A further consideration is whether our cycle 1 action plan's restriction of hot ureteroscopy to non-infected stones was itself overly conservative. A recent multicenter study by Connor et al. directly challenged this convention, comparing primary ureteroscopy within 24 h (n=29) against decompression with deferred same-admission ureteroscopy (n=15) in 44 patients with obstructing urolithiasis and confirmed concomitant infection [15]. Stone-free rate was significantly higher with primary ureteroscopy (89.7% vs. 60%, odds ratio: 5.78, p=0.029), median length of stay was shorter (three vs. eight days, p=0.038), and postoperative complication rates, including prolonged infection, were low and statistically comparable between groups (6% vs. 13%, p=0.48) [15]. The primary-ureteroscopy group had a lower mean stone size and lower ASA grade than the deferred group, reflecting the non-randomized, selectively applied nature of the intervention; some of this benefit may reflect patient selection rather than the intervention alone, but the absence of a complication signal is reassuring. This does not overturn the well-established principle that urgent decompression remains standard for patients with uncontrolled sepsis, but it suggests that a blanket exclusion of all infected cases from same-admission definitive treatment, as our department's own action plan specified, may itself be an area where practice could evolve further as local expertise and evidence develop, rather than a fixed boundary. Investment in ureteroscopy capacity is also supported on broader economic grounds as follows: a systematic review and meta-analysis by Geraghty et al. found ureteroscopy to be both clinically and financially more cost-effective than extracorporeal shock wave lithotripsy across the wider stone-treatment pathway, reinforcing the case for prioritizing ureteroscopic theater capacity over reliance on off-site or acute shock wave lithotripsy access [16].

Two findings raise governance considerations distinct from throughput. First, in cycle 1, a patient with a C-reactive protein of 121 mg/L and white cell count of 16×10^9^/L was initially managed conservatively before eventually undergoing delayed primary ureteroscopy; such inflammatory markers would ordinarily prompt consideration of urgent decompression given the risk of urosepsis in an obstructed, infected system. Second, in cycle 2, one hot ureteroscopy was performed without clear radiological evidence of an obstructing stone on updated imaging, and no stone was found at surgery. Both cases warrant individual review and illustrate that pathway metrics such as the hot ureteroscopy rate should be interpreted alongside case-level clinical governance rather than pursued as an isolated target.

The audit also identified non-evidence-based practice unrelated to surgical timing as follows: universal prescribing of tamsulosin for conservatively-managed stones, regardless of stone size or ureteric location. The large, multicenter, placebo-controlled SUSPEND trial found no significant benefit of tamsulosin or nifedipine over placebo for spontaneous stone passage, a finding reflected in current European Association of Urology (EAU) guidance, notwithstanding earlier positive signals from smaller trials summarized in the Hollingsworth meta-analysis [4-6]. While medical expulsive therapy may retain a role in selected patients, blanket prescribing across all stone sizes and locations does not reflect current best evidence and merits its own targeted intervention.

For transparency, a minor internal inconsistency is worth noting. The cycle 1 action plan estimated that 17 of the 22 surgically-managed cases (77%) would have been suitable for hot ureteroscopy, whereas the cycle 2 summary referred to an achievable rate of up to 90%. This likely reflects a difference in how candidate cases were defined between the two reviews rather than a genuine change in case mix, and would benefit from a consistent, prospectively-defined case-selection tool in future cycles.

Limitations

This was a retrospective, single-center audit relying on ward-level data and the clinical portal; documentation gaps could not be excluded, and formal statistical comparison between cycles was not performed given the relatively small sample sizes. The scope of data collection differed between cycles as follows: cycle 1 captured the entire admission cohort (48 cases), while cycle 2 captured only the surgically-managed subgroup (20 cases); comparisons are therefore presented as a proportion of surgically-managed cases in both cycles, and direct comparison of overall admission volumes or conservative-management outcomes between cycles is not possible from the data collected. Suitability for hot ureteroscopy was assessed retrospectively by case review rather than against a prospectively applied, structured selection tool, introducing potential review bias. As a two-cycle before-and-after audit without a concurrent control group, the observed improvement cannot be fully attributed to the action plan alone, although the temporal association with a specific, department-wide intervention makes this the most plausible explanation.

## Conclusions

A structured closed-loop audit, comprising a baseline cycle, an explicit department-agreed action plan, and a re-audit cycle, more than doubled the hot ureteroscopy rate for obstructing ureteric stones in our department, from 22.7% to 50% of surgically-managed cases, moving practice closer to the GIRFT (January 2022) standard. Residual missed opportunities and marked inter-surgeon variation support embedding a dedicated hot ureteroscopy theater list and a named responsible clinician, reviewing local prescribing guidance for medical expulsive therapy in light of current trial evidence, and undertaking a further audit cycle using a prospectively defined, consistent case-selection tool.
